# Clinical nurses’ moral courage and related factors: an empowerment perspective

**DOI:** 10.1186/s12912-022-01093-9

**Published:** 2022-11-23

**Authors:** Kaili Hu, Juan Liu, Lisi Zhu, Yanrong Zhou

**Affiliations:** Department of Surgery, Tongji Hospital, Tongji Medical College, Huazhong University of Science and Technology, No.1095 Jie Fang Avenue, Hankou Wuhan, 430030 P.R. China

**Keywords:** Moral courage, Nurse, Sructural empowerment, Psychological empowerment, Factors influencing

## Abstract

**Background:**

Moral courage as part of the moral competence of nurses has received increasing attention. Determination of the factors affecting moral courage is important in improving the quality of care. The purpose of this study was to investigate moral courage and related factors among frontline nurses from an empowerment perspective.

**Methods:**

A cross-sectional study was conducted using data collection instruments comprising four main parts: the self-designed form of demographic characteristics, Conditions for Work Effectiveness II (CWEQ-II), Spreitzer’s Psychological Empowerment Scale (PES) and Nurses’ Moral Courage Scale (NMCS). Data were collected from 226 nurses in a tertiary hospital between February and March 2022 in Wuhan, the capital city of Hubei Province in central China. Descriptive statistics and multiple linear regression were used to analyze the data.

**Results:**

The means of the total scores for the CWEQ-II, PES and the NMCS were 3.52 (SD = 0.69), 3.85 (SD = 0.53) and 3.90 (SD = 0.67), respectively. All the dimensions and the total scores of the CWEQ-II and PES were significantly correlated with the NMCS (*p* < 0.001). According to the multivariate stepwise regression analysis, CWEQ-II and PES were determined to be factors affecting NMCS. These variables explained 35.9% of the total variance in the moral courage scores of nurses.

**Conclusion:**

The level of moral courage among nurses is above average. Structural empowerment and psychological empowerment were the key factors affecting the promotion of moral courage. Hospital and organizational administrations should be conscious of the role of attach structural empowerment and psychological empowerment in the nursing workplace in increasing moral courage.

## Background

To provide care of high quality, nurses face several moral issues requiring them to have moral abilities in professional performance [1]. Moral courage means the courage or inner strength a person has when acting in ethical conflicts according to ethical principles and one’s own values and beliefs, even at the risk of negative outcomes for the acting individual [2]. Morally courageous acting manifested itself as speaking up and acting as the patients’ advocate when patients’ rights, safety, or good care were threatened. As a part of nurses’ moral competence, moral courage has gained increasing interest in strengthening nurses acting on their moral decisions and offering alleviation to their moral distress [3]. Moral courage has been described as a personal virtue that could become a natural part of an individual’s behavior and moral deliberation, and need to be learned and developed through training or research [4].

Most previous studies have explored the related factors and outcomes of nurses’ moral courage in concept analyses, cross-sectional and qualitative studies [3, 5–7]. Nurses’ mean score of the 21-item Nurses’ Moral Courage Scale (NMCS) was from 3.77 to 4.30 on a 5-point Likert-type scale [6–8]. Nurses perceived themselves as morally courageous, and the level of moral courage was rather high, especially when they were in a direct interpersonal relationship with their patients. In terms of influencing factors, one study of 559 nurses from two hospitals in Flanders found that age, experience, professional function, level of education and personal interest were associated with the moral courage of nurses [6]. Besides, one study reported that individual and organizational factors, such as positive personal experiences, commitment to ethical principles, supportive work environment, and teamwork, were associated with moral courage in nursing [3]. Regarding the outcome of moral courage, the findings showed that moral courage has a strong and direct correlation with moral sensitivity and safe nursing care [9].

Today, making ethical decisions that promote the safety and well-being of patients in healthcare settings is more challenging than ever [10]. Healthcare leaders who demonstrate moral courage and create environments that promote morally courageous acts would enable nurses to remain centered on the patients, families, and communities we serve [11]. As mentioned, moral courage is a virtue that can be taught, learnt, and practiced. Then, identifying its influencing factors from the perspective of organizational and personal perspectives in depth is a relevant way to further strengthen nurse’ moral courage. Therefore, this study used a cross-sectional method to investigate moral courage and related factors among frontline nurses from an empowerment perspective, which can provide directions for further assistance policies and psychological support.

Empowerment is defined as the ability to empower to synergize and enable employees to achieve excellence. Empowering nurses contributes to improving their attitudes and increasing organizational effectiveness. Empowerment includes structural empowerment and psychological empowerment, the former proposed by Kanter and the latter by Spreitzer [12]. Structural empowerment refers to the existence of social structures at work that allow individuals to achieve their work goals through access to opportunities, relevant information, support and resources [13]. Psychological empowerment refers to a state of increased intrinsic task motivation. It consists of four cognitive components: sense of meaning, competence, self-determination and impact [14].

Based on the complementary theories of Kanter and Spreiter, there is a significant relationship among nurses’ clinical behavior and structural empowerment and psychological empowerment [14]. Previous researches have investigated the independent relationship between moral courage and psychological empowerment [8]. Theoretically, based on previous research on empowerment, it is reasonable to expect that individuals who perceive a high level of empowerment possess a high level of moral courage. However, studies on structural empowerment, psychological empowerment and moral courage are evaluated separately, and the relationships among structural empowerment and moral courage have not been reported previously in the literature. Thus, the purpose of this study was to investigate the current status and relationships among moral courage, structural empowerment, and psychological empowerment. The results of this study could provide a foundation for exploring effective strategies to enhance nurses’ moral courage.

## Methods

### Study design and setting

This was a cross-sectional study and strictly followed the guidelines for reporting observational studies [15]. The study was conducted in a tertiary hospital in Wuhan, the capital city of Hubei Province in China.

### Study population and sample

The inclusion criteria were as follows: nurses who volunteering to participate in the study and having at least 1 year of experience in clinical settings. Nurses who were on vacation and came to the hospital temporarily for further study and training during data collection were excluded. The estimation of the minimum sample size required was calculated with the G*power software, version 3.1.9.6. For this calculation, it was expected to create a regression model based on a linear multiple regression analysis with a medium effect size of 0.15, an alpha equivalent to 0.05, a power (1-β) of 0.95, and at least 3 tested predictors from 11 variables analyzed. Considering 20% dropout, the required sample size in this study was set at 143 cases. Here, the sample size was equal to 226 nurses exceeding the minimum sample size.

### Data collection

It was a web-based questionnaire that used convenience sampling. Under the guidance of the research assistants, the participants could have a clear understanding of the research purpose, research process and instructions. A web-based QR code about the questionnaire was given to the participant after written consent was signed if he/she was interested in this study. A total convenience sample of 226 nurses took part in this study.

### Measurement

A self-designed form including items on gender (female and male), age, working years, marital status (single, married, divorced), number of children (none, one, two or above), education level (bachelor’s degree, master’s degree), seniority (junior, intermediate, senior), organization position (staff nurse, instructional supervision, specialist nurse, head nurse), and workplace (general ward, intensive care units) was used to collect demographic characteristics.

Structural empowerment. This study utilized the Chinese version of the CWEQ-II to measure structural empowerment [16, 17]. The CWEQ-II, which consists of 19 items, measures nurses’ perception of their access to work empowerment structures described by Kanter (information, support, resources, opportunity, formal and informal power) and ranging from 1 (none) to 5 (a lot) [16]. The subscale was obtained by summing and averaging the items. An overall empowerment scale was created by averaging the 6 subscales. A higher score indicates greater perceived structural empowerment. A two-item global empowerment scale, which is used for validation purposes, correlated positively with the CWEQ-II (*r* = 0.690, *p* < 0.001) in this study, supporting the construct validity of the instrument. The Cronbach’s alpha for the six subscales and total items in this research ranged from 0.753 to 0.943.

Psychological empowerment. PES was used to measure nurses’ psychological empowerment. Li et al. [18] translated the scale to Chinese (after obtaining permission from the inventor of the scale) and then confirmed the reliability and validity of this scale in a sample of Chinese nurses. It has 12 questions and consists of four subsections measuring meaning, self-determination, competence, and impact. This inventory uses a 5-point Likert scale (from completely disagree = 1 to completely agree = 5). All items together take their average score, and a higher score indicated higher perceived psychological empowerment [18]. In this study, Cronbach’s alpha for the dimensions and the scale ranged from 0.907 to 0.942.

Moral courage. Data were collected with the Chinese version of the NMCS [19]. Permission to use the NMCS was obtained from the copyright holder [20]. The scale consists of 21 items measuring the moral courage of nurses in four dimensions: compassion and true presence (5 items), moral responsibility (4 items), moral integrity (7 items), and commitment to good care (5 items) [20]. The NMCS was based on a Likert-type scale. The responses are based on a severity scale in the range of does not describe me at all (1) to describe me very well (5). The items are presented in a random order so they cannot be associated directly with the dimensions to which they belong. The total score for each scale is obtained by calculating the average scores of all items, which can be between 1 and 5. Higher scores indicate higher moral courage. The Cronbach’s alpha value of the total scale in the Chinese NMCS was 0.905 [19]. In this study, Cronbach’s alpha for the dimensions and the scale ranged from 0.850 to 0.967.

### Data analysis

Data were processed using SPSS 26.0 for Mac statistical software program. The demographic characteristics of the participants, CWEQ-II, PES, and NMCS, were analyzed using frequency, percentage, mean, and standard deviation (SD). The relationships between CWEQ-II, PES, and NMCS were determined using Pearson’s correlations. Stepwise multiple linear regression analysis was carried out with participants’ demographics, the CWEQ-II and PES scores as independent variables and the total NMCS score as the dependent variable to identify the factors influencing clinical nurses’ moral courage. Statistical significance was set at *p* < 0.05.

## Results

### Demographic characteristics of participants

Two hundred and twenty-six nurses participated in this study, with a response rate of 20.55%. Detailed characteristics of the participants were summarized in Table 1. All nurses were female in this study. The average age and working years were 34.54 ± 5.68 (range 23–53) and 12.40 ± 6.79 (range 1–34), respectively. Of the participants, 80.09% were married, and 75.22% had one or more children. Approximately 90% had a bachelor’s degree, while 9.29% had a master’s degree. About half of the participants were junior and half were intermediate. The majority of participants were staff nurses (77.43%). Participants whose workplace was in the general ward accounted for 87.61%.

**Table 1:** Characteristics of participants (*N* = 226)

Characteristics	Categories	n (%) or Mean (SD)
Age		34.54 (5.68)
Working years		12.40 (6.79)
Gender	Female	226 (100)
Marital status	Single	40 (17.70)
	Married	181 (80.09)
	Divorced	5 (2.21)
Number of children	None	56 (24.78)
	One	132 (58.41)
	Two or above	38 (16.81)
Education level	Bachelor’s degree	205 (90.71)
	Master’s degree	21 (9.29)
Seniority	Junior	108 (47.79)
	Intermediate	112 (49.56)
	Senior	6 (2.65)
Organization position	Staff nurse	175 (77.43)
	Instructional supervision	33 (14.60)
	Specialist nurse	10 (4.42)
	Head nurse	8 (3.54)
Ward	General	198 (87.61)
	Intensive care	28 (12.39)

### Structural empowerment, psychological empowerment and moral courage among participants

Descriptive statistics for the study variables were presented in Table 2. The mean of the total scores for the NMCS was 3.90 (SD = 0.67). The means of the total scores for the CWEQ-II and PES were 3.52 (SD = 0.69) and 3.85 (SD = 0.53), respectively. The dimensions’ mean and SD were shown in Table 2.

**Table 2:** CWEQ-II, PES and NMCS dimensions’ mean and SD (*N* = 226)

Variables	Mean	SD
CWEQ-II		
Opportunity	3.62	0.78
Information	3.26	0.95
Support	3.96	0.82
Resources	3.65	0.84
Formal power	3.20	0.91
Informal power	3.47	0.80
Global empowerment	3.93	0.70
Total	3.52	0.69
PES		
Meaning	4.26	0.56
Self-determination	3.89	0.71
Competence	4.13	0.59
Impact	3.13	0.89
Total	3.85	0.53
NMCS		
Moral integrity	3.97	0.68
Commitment to good care	3.72	0.74
Compassion and true presence	3.98	0.72
Moral responsibility	3.90	0.72
Total	3.90	0.67

*CWEQ-II *Conditions for Work Effectiveness-II, *PES *Psychological Empowerment Scale, *NMCS *Nurses’ Moral Courage Scale

### Correlations among structural empowerment, psychological empowerment and moral courage

As the correlations were shown in Table 3, all the dimensions and the total scores of the CWEQ- II and PES were significantly correlated with the NMCS. Highly authorization both in structural and psychological correlated with higher levels of moral courage.

**Table 3:** Correlation coefficients among NMCS and CWEQ- II, PES (*N* = 226)

Variables	Moral integrity	Commitment to good care	Compassion and true presence	Moral responsibility	NMCS
CWEQ-II	0.516**	0.486**	0.538**	0.513**	0.541**
Opportunity	0.329**	0.320**	0.364**	0.328**	0.353**
Information	0.416**	0.386**	0.436**	0.423**	0.437**
Support	0.415**	0.335**	0.460**	0.380**	0.421**
Resources	0.384**	0.322**	0.394**	0.385**	0.391**
Formal power	0.433**	0.427**	0.443**	0.435**	0.458**
Informal power	0.496**	0.523**	0.490**	0.503**	0.530**
Global empowerment	0.413**	0.362**	0.371**	0.441**	0.417**
PES	0.544**	0.555**	0.551**	0.592**	0.588**
Meaning	0.446**	0.373**	0.466**	0.424**	0.452**
Self-determination	0.366**	0.377**	0.412**	0.439**	0.415**
Competence	0.577**	0.538**	0.567**	0.592**	0.599**
Impact	0.330**	0.418**	0.305**	0.389**	0.377**

**P < 0.001

### Influencing factors of moral courage among participants

The results showed that participants’ moral courage was affected by structural empowerment and psychological empowerment (Table 4). These variables explained 35.9% of the total variance in the moral courage scores of nurses.

**Table 4:** Variables related to NMCS (multivariate stepwise regression analysis, *N* = 226)

Variables	B	Standard error	β	t	p
Constant	1.010	0.330	-	3.061	0.002
CWEQ- II	0.247	0.078	0.251	3.155	0.002
PES	0.488	0.105	0.381	4.636	< 0.001

*R* = 0.632, adjusted *R*^2^ = 0.359, *F* = 10.007, *P* < 0.001

## Discussion

This study aimed to evaluate the current status of moral courage among nurses in central China and explore factors influencing it from an empowerment perspective. The level of the total score of moral courage was above average in this study. There was a positive correlation between structural empowerment, psychological empowerment and moral courage. The level of moral courage experienced by clinical nurses was also influenced by structural empowerment and psychological empowerment.

In our sample, the average NMCS score was 3.90 ± 0.67, between the scores of Konings et al.’s (mean, 3.77 ± 0.537) [6] and Khoshmehr et al.’s (mean, 4.30 ± 0.503) [8] findings. This result may be related to the differences between measurements conducted in different cultural contexts, and also suggests that clinical nurses’ moral courage in this hospital behaves generally well. It is worth noting that the response rate in this study was 20.55%, so that we do not know the level of moral courage of the remaining nurses (about 80%). The response rate of a survey provides an indication of response bias and the consequent representativeness of the results of the study. Therefore, the nonresponse bias may exist in this study, that is systematic variation in a measure of importance to the study, between those who respond to a survey and those who do not [20]. Whilst there is no evidence of an ideal response rate relationship to survey validity, response rates can be enhanced by including monetary incentives, and repeat contact with non-responders in future studies.

The average scores of items on the dimensions of “moral integrity” and “compassion and true presence” were moderately higher than those on the dimensions of “commitment to good care” and “moral responsibility” (Table 2), which was in line with another study in Finland [6]. Moral integrity focuses on adhering to the principles and values of the profession and healthcare in general, particularly in situations where taking the risk of negative consequences from others is a possibility, thus focusing on the very core of moral courage [21]. This finding reflects the fact that the teachings of Confucius, as principles for social interaction, have a great influence on Chinese nurses’ behavior. Confucianism is based on the principles of loyalty and encouraging harmony and altruism, which is related to the fulfillment of duty and the utmost commitment to it, coupled with impartiality in decision-making [22]. Compassion and true presence describe care situations in which encountering the patient’s vulnerability in sickness and suffering demands that the nurse overcome her or his own inner fears, forcing the nurse to encounter her or his own vulnerability to be able to act courageously [21]. Altruism may also be one reason to explain the findings of this study, consistent with Wang et al.’s study in central China [23].

In this study, the total score and the dimensions of the NMCS positively correlated with the CWEQ-II and PES (Table 3, *p* < 0.001). This finding suggests that empowerment can significantly affect the moral courage of nurses and was consistent with one previous research finding [8]. The mean scores of structural empowerment and psychological empowerment of participants were above average, which is consistent with the results of some earlier studies [24, 25]. Empowerment in the nursing literature was described as something that nurses did for their patients or as an individual process of self-awareness and actualization [26]. In addition, hierarchy in organizations can inhibit moral courage [27]. Highly empowered groups can create a more flexible work atmosphere for the achievement of work targets [28]. Accordingly, empowerment has important contributions to medical care and workers. In addition, empowerment is closely related to the benefits of the practice environment such as increased retention and job satisfaction, improved and safer care [29]. Therefore, when pressured to conform to unethical or outdated practices, empowered nurses will overcome their fears, endure the consequences and act in a manner consistent with their professional values, which is the core ideology of moral courage.

The regression analysis showed that CWEQ-II and PES played significant roles in the NMCS (Table 4, *p* < 0.05). As the level of structural empowerment and psychological empowerment increased, the moral courage level of nurses improved. This may suggest that there may be an optimum combination of research variables to enforce moral courage. Previous study also found that nurses would experience moral distress when they feel disempowered or impeded in taking the ethically right course of action [30]. This is because that the environment of nurse practice strongly influences ethics norms and social practice, and structural empowerment is an important aspect of the practice environment [31]. In addition, the benefits of structural empowerment with the content of opportunity, support, information, resources, formal power and informal power, can be manifested in improved employees’ attitudes and progress toward meeting organizational goals, in which nurses’ attitudes of their power to resolve ethical problems are essential to the decision of whether to take action and that empowerment strategies can lead to ethical action [13]. Moreover, positive outcomes such as collaborations and trust are the result of providing improved authorization level within the work environment, which can itself be the cornerstone for establishing a positive moral atmosphere. However, interventions to improve delegation were limited in the published literature. Therefore, nursing managers can enhance moral courage by creating a clinical environment that facilitates obtaining the support, information, and resources needed for nurses to perform their duties effectively; recognizing nurses’ achievements; providing opportunities for learning and professional development; and expanding their responsibilities, so that nurses can present greater moral courage for patients’ outcome.

On the other hand, the significant relationship between psychological empowerment and self-assessed moral courage in Table 4 supports the importance of personal feelings in acting morally courageously. In a study in Iran by Khoshmehr et al. [8] also concluded that nurses’ moral courage could be enhanced by reinforcing their psychological empowerment, which led to increased patient satisfaction and quality care. Psychological empowerment refers to one’s perceptions about themselves in relationship to one’s work environment. The four cognitive factors of psychological empowerment with meaning, competence, self-determination and impact reflect an active orientation and feeling of control toward work [32]. Hence, experiencing empowerment and intrinsic motivation can result in positive forms of work performance, including acting courageously on ethical issues. Nurses’ choices, efforts, and determination to cope with and solve ethical dilemmas depend on their own ethical competence [33]. It is noteworthy that ethics education can be effective in improving knowledge, perception, confidence, and ethical behavior [34]. Robinson et al. [35] developed an educational program, the Clinical Ethics Residency for Nurses, to strengthen nurses’ moral agency, that is, an enhanced ability to act to bring about change. This program employed a variety of methods based on adult learning theory, such as the active application of ethics knowledge to patient scenarios in classroom discussion, simulation, lecture-style classes and the clinical practicum. Indeed, being aware of competence or self-determination in a clinical setting can provide a basis for understanding and improving nurses’ moral behaviors. This finding emphasized that further attention needs to cover strategies for psychological empowerment to encourage nurses to demonstrate moral courage when confronted with ethical misconduct.

There were several limitations that need to be acknowledged. First, we only conducted the investigation in one hospital, and convenience sampling was used, so selection bias may have occurred. It is remarkable that we did not identify a significant relationship between moral courage and demographic features, which may be due to the sampling method. Second, since the study sample is small, it is not possible to evaluate a proposed model in which nurses’ psychological empowerment intermediate the association between nurses’ structural empowerment and moral courage. Therefore, further research is required to analyze and explore the direct and indirect effects of variables on moral courage with a more rigorous study design and larger samples from other nursing fields.

## Conclusion

The level of moral courage among nurses in central China is above average. This level of moral courage by Chinese-registered nurses was influenced by structural empowerment and psychological empowerment. Strategies to enhance moral courage in nurses should incorporate interventions that accurately improve the degree of authorization, whether in structural empowerment or psychological empowerment. There is an urgent need to develop and test such interventions and identify whether enhancing empowerment leads to improved levels of moral courage. Our findings also suggest that hospital and organizational administrations should intensify ethics education to improve nurses’ moral courage.

## Data Availability

The datasets used and analyzed during the current study are available from the corresponding author on reasonable request.

## Abbreviations

CWEQ-II: Conditions for Work Effectiveness II
PES: Psychological Empowerment Scale
NMCS: Nurses’ Moral Courage Scale

## References

[1] Amiri E, Ebrahimi H, Vahidi M, Asghari JM, Namdar AH (2019). Relationship between nurses’ moral sensitivity and the quality of care. Nurs Ethics.

[2] Pajakoski E, Rannikko S, Leino-Kilpi H, Numminen O (2021). Moral courage in nursing - An integrative literature review. Nurs Health Sci.

[3] Numminen O, Konings K, Claerhout R, Gastmans C, Katajisto J, Leino-Kilpi H (2021). Validation of the Dutch-language version of Nurses’ Moral Courage Scale. Nurs Ethics.

[4] Eleni Papouli (2019). Aristotle’s virtue ethics as a conceptual framework for the study and practice of social work in modern times. Eur J Social Work.

[5] Numminen O, Repo H, Leino-Kilpi H (2017). Moral courage in nursing: A concept analysis. Nurs Ethics.

[6] Konings KJ, Gastmans C, Numminen OH, Claerhout R, Aerts G, Leino-Kilpi H (2022). Measuring nurses’ moral courage: an explorative study. Nurs Ethics.

[7] Hauhio N, Leino-Kilpi H, Katajisto J, Numminen O (2021). Nurses’ self-assessed moral courage and related sociodemographic factors. Nurs Ethics.

[8] Khoshmehr Z, Barkhordari-Sharifabad M, Nasiriani K, Nasiriani K, Fallahzadeh H (2020). Moral courage and psychological empowerment among nurses. BMC Nurs.

[9] Khodaveisi M, Oshvandi K, Bashirian S, Khazaei S, Gillespie M, Masoumi SZ (2021). Moral courage, moral sensitivity and safe nursing care in nurses caring of patients with COVID-19. Nurs Open.

[10] Salmela S, Koskinen C, Eriksson K (2017). Nurse leaders as managers of ethically sustainable caring cultures. J Adv Nurs.

[11] Edmonson C (2015). Strengthening Moral Courage Among Nurse Leaders. Online J Issues Nurs.

[12] Cicolini G, Comparcini D, Simonetti V (2014). Workplace empowerment and nurses’ job satisfaction: a systematic literature review. J Nurs Manag.

[13] Fragkos KC, Makrykosta P, Frangos CC (2020). Structural empowerment is a strong predictor of organizational commitment in nurses: A systematic review and meta-analysis. J Adv Nurs.

[14] Zhang X, Ye H, Li Y (2018). Correlates of structural empowerment, psychological empowerment and emotional exhaustion among registered nurses: A meta-analysis. Appl Nurs Res.

[15] von Elm E, Altman DG, Egger M, Pocock SJ, Gøtzsche PC, Vandenbroucke JP (2014). The Strengthening the Reporting of Observational Studies in Epidemiology (STROBE) Statement: guidelines for reporting observational studies. Int J Surg.

[16] Laschinger HK, Finegan J, Shamian J (2001). Promoting nurses’ health: Effect of empowerment on job strain and work satisfaction. Nurs Econ.

[17] Jia T. (2007). Investigation of the influencing effect of work empowerment on clinical nurses’ self-efficacy and job-control (MD Thesis, Sichuan university). https://kns.cnki.net/kcms/detail/detail.aspx?dbcode=CMFD&dbname=CMFD2008&filename=2007220619.nh&uniplatform=NZKPT&v=xVZYGjyltKgFKmTgTivDVWQ_DkB09EJj2kt7RMtN8FanvXWvs9VNVLhTg1aGcULK.

[18] Li CP, Li XX, Shi K, Chen XF (2006). Psychological empowerment: measurement and its effect on employee work attitude in china. Acta Physiol sinica.

[19] Wang SY, Wei LL, Zhang Y, Liu T, Jiang WB, Yang HP (2019). The reliability and validity of the Chinese version Nurses’ Moral Courage Scale. J Nurs Sci.

[20] Sammut R, Griscti O, Norman IJ (2021). Strategies to improve response rates to web surveys: A literature review. Int J Nurs Stud.

[21] Numminen O, Katajisto J, Leino-Kilpi H (2019). Development and validation of Nurses’ Moral Courage Scale. Nurs Ethics.

[22] Badanta B, González-Cano-Caballero M, Suárez-Reina P, Lucchetti G, de Diego-Cordero R. How Does Confucianism Influence Health Behaviors, Health Outcomes and Medical Decisions? A Scoping Review. J Relig Health. Epub ahead of print 10 Feb 2022. 10.1007/s10943-022-01506-8. 10.1007/s10943-022-01506-8 PMC9314298 35141796

[23] Wang H, Liu Y, Hu K, Zhang M, Du M, Huang H (2020). Healthcare workers’ stress when caring for COVID-19 patients: An altruistic perspective. Nurs Ethics.

[24] Khan BP, Quinn Griffin MT, Fitzpatrick JJ (2018). Staff Nurses’ Perceptions of Their Nurse Managers’ Transformational Leadership Behaviors and Their Own Structural Empowerment. J Nurs Adm.

[25] Sahraei Beiranvand M, Beiranvand S, Beiranvand S, Mohammadipour F (2021). Explaining the effect of authentic and ethical leadership on psychological empowerment of nurses. J Nurs Manag.

[26] Friend ML, Sieloff CL (2018). Empowerment in Nursing Literature: An Update and Look to the Future. Nurs Sci Q.

[27] Kleemola E, Leino-Kilpi H, Numminen O (2020). Care situations demanding moral courage: Content analysis of nurses’ experiences. Nurs Ethics.

[28] Mudallal RH, Othman WM, Al Hassan NF (2017). Nurses’ Burnout: The Influence of Leader Empowering Behaviors, Work Conditions, and Demographic Traits. Inquiry.

[29] Kennedy S, Hardiker N, Staniland K (2015). Empowerment an essential ingredient in the clinical environment: a review of the literature. Nurse Educ Today.

[30] DiGangi Condon KA, Berger JT, Shurpin KM (2021). I’ve Got the Power: Nurses’ Moral Distress and Perceptions of Empowerment. Am J Crit Care.

[31] García-Sierra R, Fernández-Castro J (2018). Relationships between leadership, structural empowerment, and engagement in nurses. J Adv Nurs.

[32] Zhou H, Chen J (2021). How Does Psychological Empowerment Prevent Emotional Exhaustion? Psychological Safety and Organizational Embeddedness as Mediators. Front Psychol.

[33] Obeid S, Man M (2020). Strengthening Perceptions of Ethical Competence Among Nursing Students and Graduates. SAGE Open Nurs.

[34] Laabs CA (2012). Confidence and knowledge regarding ethics among advanced practice nurses. Nurs Educ Perspect.

[35] Robinson EM, Lee SM, Zollfrank A, Jurchak M, Frost D, Grace P (2014). Enhancing moral agency: clinical ethics residency for nurses. Hastings Cent Rep.

